# Impact of a bottom-up community engagement intervention on maternal and child health services utilization in Ghana: a cluster randomised trial

**DOI:** 10.1186/s12889-019-7180-8

**Published:** 2019-06-21

**Authors:** Robert Kaba Alhassan, Edward Nketiah-Amponsah, Martin Amogre Ayanore, Agani Afaya, Solomon Mohammed Salia, Japiong Milipaak, Evelyn Korkor Ansah, Seth Owusu-Agyei

**Affiliations:** 1 Department of Public Health Nursing, School of Nursing and Midwifery, University of Health and Allied Sciences Ho, PMB 31 Volta Region Ho, Ghana; 2 Department of Economics, University of Ghana, Legon, Accra Ghana; 3 Department of Family and Community Health, School of Public Health, University of Health and Allied Sciences, Hohoe, Ghana; 4 Department of Nursing, School of Nursing and Midwifery, University of Health and Allied Sciences, Ho, Ghana; 5 Institute of Health Research (IHR), University of Health and Allied Sciences, Ho, Ghana

**Keywords:** Clients, Community engagement, Cluster randomised trial, Ghana, Intervention, Primary healthcare, Utilization

## Abstract

**Background:**

Ghana is among African countries not likely to achieve the Sustainable Development Goal (SDG) three (3) target of reducing maternal mortality to 70 per 100,000 live births by the year 2030 if maternal and child health services utilization are not improved. Community engagement in health is therefore advocated to help address this challenge. This study evaluated the impact of a community engagement intervention on maternal and child health services utilization in Ghana.

**Methods:**

This study was a cluster randomised trial among primary healthcare facilities (*n* = 64) in the Greater Accra and Western regions in Ghana. Multivariate multiple regression analysis and paired-ttest were used to determine impact of the community engagement intervention on maternal and child health indicators at baseline and follow-up.

**Results:**

Intervention health facilities recorded significant improvements over control facilities in terms of average spontaneous vaginal deliveries per month per health facility (baseline mean = 15, follow-up mean = 30, *p* = 0.0013); child immunizations (baseline mean = 270, follow-up mean = 455, *p* = 0.0642) and female condoms distribution (baseline mean = 0, follow-up mean = 2, *p* = 0.0628). Other improved indicators in intervention facilities were average number of Human Immunodeficiency Virus (HIV) tests for non-pregnant women (baseline mean = 55, follow-up 104, *p* = 0.0213); HIV tests for pregnant women (baseline mean = 40, follow-up mean = 119, *p* = 0.0067) and malaria tests (baseline mean = 43, follow-up mean = 380, *p* = 0.0174). Control facilities however performed better than intervention facilities in terms of general laboratory tests, voluntary counselling and testing, treatment of sexually transmitted infections, male child circumcisions and other minor surgical procedures.

**Conclusion:**

Community engagement in health has the potential of improving utilization of maternal and child health services. There is the need for multi-stakeholder dialogues on complementing existing quality improvement interventions with community engagement strategies.

**Electronic supplementary material:**

The online version of this article (10.1186/s12889-019-7180-8) contains supplementary material, which is available to authorized users.

## Background

According to the World Health Organization (WHO), approximately 830 women die every day from preventable causes related to pregnancy and child birth and about 99% of all maternal deaths occur in developing countries mostly in Africa [1]. Maternal mortality per 100,000 live births in Ghana is 319 compared to the African average of 542 [2]. Nonetheless Ghana is not likely to achieve the Sustainable Development Goal (SDG) three (3) target of reducing maternal morality to 70 per 100,000 live births by the year 2030 if maternal and child health services utilization are not improved [1]. In Ghana, access to maternal and child health services is impeded by longer travel times to health facilities. For instance, 6 in 10 households which had a maternal death travelled for at least 30 min to reach the nearest health facility for maternal health services. Moreover, socio-economic limitations including poverty hinder financial access to maternal and child health services (i.e. only 15% of women in Ghana have health insurance coverage that requires no payment for drugs and services) [2].

In light of these maternal and child health challenges, many countries in Africa, including Ghana, have increasingly emphasized the need for bottom-up community engagement in the planning and implementation of healthcare services. This approach has the potential to promote utilization of safer maternal and child healthcare services [1, 3]. Ghana’s flagship Community-based Health Planning and Services (CHPS) programme is evident of the premium placed on community engagement in health to promote ownership of healthcare interventions in line with the Alma Ata declaration on primary healthcare in 1978.

Additionally, section four of the Alma Ata declaration emphasized that people have the right and duty to participate individually and collectively in the planning and implementation of their health [4]. However, four decades after the Alma Ata declarations were made full complement of these declarations are yet to be realised in many health systems in Africa with respect to the impact on maternal and child health outcomes. Empirical studies have advocated community-based health interventions as a leverage to improving access to maternal and child. Community-based health interventions have proved to be effective towards promoting acceptability and utilization of health services [5–8].

The paper evaluated the impact of a bottom-up community engagement intervention on utilization of selected maternal and child healthcare services in 64 primary healthcare facilities in Ghana after nearly two years of implementation. Service components evaluated before and after the intervention included facility-based spontaneous vaginal deliveries (SVDs), child immunizations, condom distributions, Human Immunodeficiency Virus (HIV) testing, male child circumcisions and other minor surgical procedures.

## Methods

### Study design

This was a cluster randomised trial in 64 primary health facilities (32 intervention and 32 control) and their catchment area, as detailed in previous related publications by the lead author [5–8]. Primary health facilities in this context are clinics and health centres as per the Ghana Health Service (GHS) categorizations and pyramidal levels of healthcare.

### Eligibility criteria

Private and public health facilities categorized as clinics or health centres were considered eligible for inclusion in this study. Moreover, health facilities credentialed by the National Health Insurance Authority (NHIA) in Ghana were included in the study. Health facilities with same or similar NHIA credentialed scores were included to ensure homogeneity among the control and intervention facilities. The NHIA is an agency under the Ministry of Health (MoH) established in 2003 by Parliamentary Act (650) and amended Act (852) in 2012. The NHIA is mandated to implement the NHIS, determine scheme membership contributions, registration, issuance of membership cards and regulation of private health insurance schemes in Ghana.

### Study population and setting

This study setting and population are the same as previous publications by the lead author using the same research design and approach (see Alhassan et al. [5–8]). The study was thus conducted in the Greater Accra and Western regions of Ghana in 16 administrative districts. Greater Accra region is a coastal region which also hosts the capital of Ghana (Accra); it is predominantly urban and cosmopolitan with a population of about 4 million people [7, 9]. Out of the nearly 3593 credentialed health facilities in Ghana in 2018, a total of 419 were in Greater Accra region [7, 9]. Western region which is also a coastal region has a population of a little over 2 million with 439 NHIS-credentialed health facilities as at 2018 [9]. In both regions over 50% of the credentialed facilities are primary level facilities (i.e. clinics and health centres).

### Randomisation and sampling procedure

Randomisation and sampling procedure for this study are same as previous publications by the lead author [5–8] where the sample frame entailed list of primary health facilities credentialed by the NHIA. Primary health facilities were purposively selected for this study because they are relatively less complex in terms of health service delivery and could easily be monitored for impact of the implemented intervention [7]. Moreover, these cadres of health facilities are closer to their communities in terms of service delivery and are often the first port of call in terms of formal healthcare delivery [7].

The cluster randomization involved randomly sampling eight (8) NHIS-district offices, analogous to administrative districts, from each of the two regions same as the approach used in Alhassan et al. [7]. The focus was on districts which had NHIS membership enrolment and NHIS credentialed health facilities at the time of conducting the study. NHIS districts were selected because these districts were analogous to the administrative districts in the two regions at the time of conducting the study. Also, the study focused on all facilities accredited by the NHIA at the time of conducting this study hence, the need to consider districts which had NHIS membership enrolment and NHIS accredited health facilities [5–8].

Next, four (4) health facilities were randomly picked without replacement as cluster sites from each district; two (2) facilities were then randomly assigned to intervention and control groups, making a total of 32 intervention and 32 control facilities (see Additional file 1). Prior to the randomization, Principal Component Analysis (PCA) was performed on the NHIA credentialed data in all the sixteen (16) districts to select the most homogeneous health facilities. The PCA scores allowed for comparability in the 64 sampled facilities (32 from each region), prior to randomization into control and intervention groups [5–8]. A profile of health facilities involved in the cluster randomised trial is detailed in Table 1.

**Table 1:** Profile of health facilities involved in cluster randomized trial *n* = 64

Health facilities	Baseline (Before interventions in 2012)		Follow-up (After Interventions in 2014)	
	Intervention (*n* = 32)	Control (*n* = 32)	Intervention (*n* = 32)	Control (*n* = 32)
Facility ownership	f (%)	f (%)	f (%)	f (%)
Private	14 (22)	16 (25)	13 (20)^a^	17 (27)
Public/Government	14 (22)	12 (19)	14 (22)	11 (17)
NGO/Faith Based	4 (6)	4 (6)	4 (6)	4 (6)
Total	32 (50)	32 (50)	31 (48)^a^	32 (50)
Facility location				
Urban	15 (23)	15 (23)	15 (23)	15 (23)
Rural	14 (22)	10 (16)	13 (20)^a^	9 (14)
Peri-urban	3 (5)	7 (11)	3 (5)	8 (13)
Total	32 (50)	32 (50)	31 (48)^a^	32 (50)

Source: Field Data Greater Accra and Western Regions (2014); Legend: ^a^Attrition of one health facility which was rural by location and private by ownership; f (Frequency)

### Overview of the systematic community engagement (SCE) intervention

Detailed description of the SCE intervention has been published in previous articles by the lead author [5–8]. Nonetheless, an overview of the SCE intervention is presented in this paper for the purposes of emphasis. The community engagement intervention was implemented in 32 primary health facilities for nearly one year (from June, 2013 to March, 2014) and evaluated over three months [7]. Baseline study was conducted in 2012 and the follow-up conducted in 2014 after the intervention implementation. The bottom-up intervention involved using existing community groups/associations to identify service delivery gaps in healthcare facilities through a systematic community engagement process (see Additional file. 2) [7]. Comprehensive reports on the community engagement intervention have been published by Alhassan et al. [5–8].

The SCE intervention was implemented using a bottom-up approach to promote community participation in the healthcare quality improvement value chain guided by predetermined healthcare quality proxies. The first step of the engagement process involved recruitment and training of 52 facilitators, and identification of existing community groups/associations. As part of the engagement process, one facilitator was assigned to each of the 52 community groups in the two study regions, thus 26 in each region [7].

The second step of the intervention entailed evaluation of the quality of healthcare services in the intervention health facilities by community members. The assessment was done based on the clients’ most recent encounter with the service providers, in the last six (6) months [7]. Healthcare quality proxies assessed by the community members were non-technical components of service delivery such as staff attitude towards clients, staff punctuality to work, feedback from staff to clients on their health conditions, staff providing right information to clients during service delivery and ability of health facility to dispense all prescribed medications to clients. As part of the assessment process, community members were expected to express their satisfaction or disappointment with these healthcare quality proxies. The community scoring was guided by “cartoon illustrated” five-point Likert Scale score card that ranged from 1 = “Very disappointing” to 5 = “Very Satisfactory”. Assessment scores by community members are published in Alhassan et al. [8]; besides, the focus of this paper is on impact of the community engagement activities on utilization of maternal and child health services in health facilities that benefited from the implemented interventions.

The third step of the intervention implementation involved a validation of the community members assessment scores with the relevant stakeholders such as health managers, NHIS managers, clients and traditional authorities. The validation and feedback sessions were held separately in the regional capitals of the two regions. The aim was to enable service providers address identified service quality gaps and agree with clients and other stakeholders on timelines for addressing these gaps [7].

The fourth step was a follow-up on the intervention health facilities by community facilitators also called “community quality care champions” to ensure action plans were implemented by health managers in the intervention health facilities as agreed during the validation and feedback sessions. The follow-up was done by the “community quality care champions” approximately three (3) months after the third implementation step [7].

The final step involved recognition of health facilities which were perceived by community members to have improved in the quality of their services to clients after the series of engagements with community members. The community members adjudged the best health facilities themselves to promote transparency, ownership and accountability of health providers to clients. Reward for best health facilities in service quality was an inscription of recognition displayed at the outpatient department (OPD) of the facility in addition to a cash amount of approximately US$ 280 given in Ghana Cedis (GHC) equivalence. The aim of this recognition was to stimulate health competition among peer health facilities to render client-centered quality services. See details of all the intervention implementation steps in Additional file 3 and in Alhassan et al. [7].

### Data collection instrument

Data was collected using a tool called Situational Analysis Plus (SA^+^) which has four main sections namely: Facility Information, Access to Care, Clinic Activities/Services, and Personnel/Vacancies. SA^+^ was developed by the PharmAccess Foundation and SafeCare Initiative in the Netherlands and has been tested in a number of African countries including Ghana. Details of the SA^+^ tool can be found in Alhassan et al. [5].

### Statistical analysis

Only data from primary health facilities contacted at baseline and follow-up was used for the final analysis in line with the protocol for evaluating effectiveness of interventions [10]. STATA statistical software version 12.0 (StataCorp, College Station. Texas USA) was used for all analysis. Multivariate multiple regression analysis and paired t-test were used to determine true impact of the community engagement intervention on the key outcome variables of interest after testing for multicollinearity and controlling effects of relevant covariates.

### Outcome variables

Main outcome variables were: number of spontaneous vaginal deliveries; number of child immunizations conducted; number of female condoms distributed; number of HIV tests for non-pregnant women, number of HIV tests for pregnant women, and number of malarial tests. These outcome variables were recorded on “per month per health facility” basis.

## Results

### Background information of primary health facilities

Baseline study was conducted between March and June, 2012 while the follow-up data was collected between July and October, 2014 after the intervention implementation which lasted for nearly a year (i.e. June, 2013 to March, 2014). At baseline, a total of 64 primary health facilities participated in the study. This number included 30 private-for-profit, 26 government owned and 8 faith-based facilities. At follow-up the number of facilities reduced marginally to 63 due to attrition comprising of 30 private-for-profit, 25 government-owned and 8 faith-based facilities (see Table 1).

Results from the baseline data showed that in 2012, the average number of clients accessing outpatient and inpatient services were 1011(SD = 787) compared to an average of 1317 (SD = 157) patients in 2014 at follow-up. The number of clients accessing inpatient and outpatient services increased in the intervention facilities from an average of 952 (SD = 692) at baseline (2012) to 1453 (SD = 1329) at follow-up (see Table 2).

**Table 2:** Profile of clients accessing care in intervention and control health facilities

	Baseline (2012)				Follow-up (2014)			
Variables		Intervention	Control	Mean Diff		Intervention	Control	Mean Diff
Total clinic attendance	Obs.	Mean (SD)*	Mean (SD)*	Mean*	Obs.	Mean (SD)*	Mean (SD)*	Mean*
OPD/IPD clients	64	952 (692)	1071 (880)	−120	63	1453 (1329)	1185 (1165)	268
Gender disaggregation								
Male clients	64	918 (664)	1056 (862)	− 138	63	432 (387)	359 (328)	73
Female clients	64	609 (427)	673 (566)	−64	63	1021 (981)	827 (868)	195
Age disaggregation								
Under 5 years	46	181 (158)	232 (235)	−51	63	291 (343)	280 (330)	10
5 years^+^	46	135 (183)	103 (99)	32	63	299.7 (388)	192 (166)	107
Income groups of clients								
Very low income	64	19 (19)	20 (22)	0.72	63	17.2 (13.7)	16.6 (13.7)	0.66
Low income	64	21 (12)	21 (13)	0.22	63	43.48 (25.5)	34.75 (23.6)	8.73
Middle income	64	18 (8)	21 (14)	−3	63	28.9 (23.8)	35.3 (23.9)	−6.3
High income	64	42 (23)	38 (24)	4	63	10.35 (14)	13.4 (18.5)	−3.05
Clinic efficiency								
Average bed occupancy rate	64	32.4%(27.9)	32.5%(26.5)	−0.09	63	32.4%(26.7)	25.1%(22.0)	7.26
Average length of inpatient stay	64	1.75 (2.5)	1.63 (1.5)	0.125	38	1.194 (0.60)	1.70 (2.3)	0.51

Source: Field Data Greater Accra and Western Regions (2014)

Legend: *All means and standard deviations have been rounded up to the nearest decimal point; SD (Standard Deviation)

The results further revealed an overall reduction in bed occupancy rate in intervention and control facilities over time. Intervention facilities at baseline recorded an average of 34% (SD = 28) per month per health facility while control facilities recorded 33% (SD = 27) per month per health facility. At follow-up, intervention facilities recorded an average bed occupancy rate of 32% (SD = 27) per month per health facility while control facilities recorded 25% (SD = 22) per month per health facility. Average length of inpatient stay at baseline was approximately 2 days (SD = 2.0) compared to 1 day (SD = 1.7) at follow-up in intervention and control facilities (see Table 2).

### Contextual factors: human and material resources in sampled primary health facilities

**Fig. 1:**
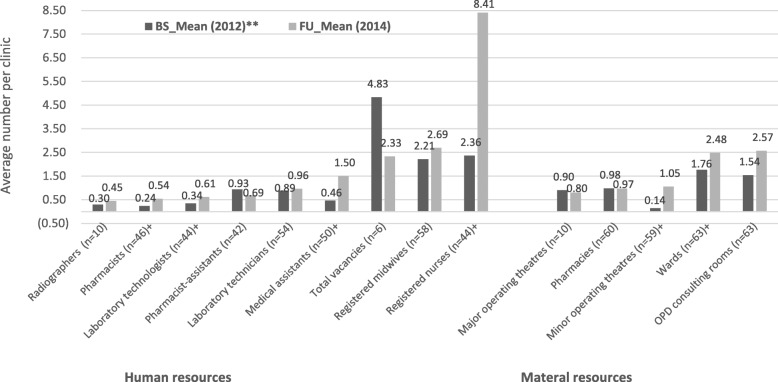
Human and material resource capacity of health facilities. Source: Field Data Greater Accra and Western Regions (2014). Legend: **All means have been rounded up to the nearest decimal point; +Independent t-test statistically significant at 95% confidence level (*p* < 0.05); BS (Baseline); FU (Follow-up)

There were contextual factors existing in the intervention and control facilities which were independent of the intervention. These were typically material and human resources of the facilities. Overtime, there was an increase in the average number of staff per health facility. During the follow-up survey, some improvement was observed in the number of medical assistants, pharmacists, laboratory technologists, and registered nurses working in both intervention and control facilities. Even though the number of other professional categories also increased, the differences between the baseline and follow-up figures were not statistically significant (see Fig. 1). These were contextual factors which were outside the sphere of control of the study and the intervention might not have influenced their outcome.

In terms of the material resources of intervention and control facilities over time, the average number of wards in a health facility increased marginally from an average of 1.8 at baseline to an average of 2.4 (*p* < 0.001) at follow-up. Likewise, average number of minor surgical operating theatres per clinic increased marginally from 0.14 at baseline to 1.05 at follow-up (p < 0.001) (see Fig. 1). As stated earlier, these variations might not have been necessarily influenced by the intervention since they were outside the sphere of control of this study.

### Maternal and child health service utilization and their correlates

On the average, the number of spontaneous vaginal deliveries per month per clinic increased from 15 (SD = 18) at baseline to 30 (SD = 33) at follow-up, *p* = 0.0013 in intervention facilities relative to a marginal increase from 22 (SD = 16) at baseline to 31 (SD = 26) at follow-up in control facilities. Similarly, the number of child immunizations conducted in intervention facilities per month per health facility increased from 270 (SD = 290) at baseline to 455(SD = 463) at follow-up compared to 287 (SD = 361) at baseline and 307 (SD = 258) at follow-up in control facilities.

Other improvements observed in intervention facilities at follow-up were the number of female condoms distribution. Among the 5 intervention facilities that rendered female condom services, no female condom was distributed at baseline but increased to an average of 2 (SD = 2) per facility per month at follow-up. Among control facilities that distributed female condoms, the average number distributed per facility per month increased from 0 at baseline to 6 (SD = 10) at follow-up.

Among the 24 intervention health facilities that run tests for HIV, an average of 55 (SD = 65) tests were conducted for non-pregnant clients per facility per month at baseline compared to 104 (SD = 81) tests per facility per month at follow-up, *p* = 0.0213. Even though in control facilities there was marginal increase in HIV tests for non-pregnant women, it was not significant. In the case of HIV tests for pregnant women, an average of 40 (SD = 43) tests per facility per month was recorded in intervention facilities at baseline but increased to 119 (SD = 128) per facility per month at follow-up (*p* = 0.0067). In control facilities, although some increases were recorded, they were marginal and statistically insignificant (see Table 3).

**Table 3:** Health service utilization in intervention and control health facilities

Service Components	Intervention				Control			
	Obs.	Baseline Data (2012)	Follow-up Data (2014)		Obs.	Baseline Data (2012)	Follow-up Data (2014)	
Maternal healthcare services		Mean (SD)^a^	Mean (SD)^a^	*p*-value		Mean (SD)^a^	Mean (SD)^a^	*p*-value
Normal deliveries	25	15 (18)	30 (33)	0.0013^**^	19	22 (16)	31 (26)	0.0586
Antenatal services	24	138 (150)	183 (206)	0.1113	21	148 (166)	181 (181)	0.3325
Child healthcare services								
Immunizations	15	270 (290)	455 (463)	0.0642	18	287 (361)	307 (258)	0.7899
Family planning services								
Distributed male condoms	11	117 (211)	67 (94)	0.4948	14	68 (91)	24 (35)	0.1228
Distributed female condoms	5	0 (0)	2 (2)	0.0628	9	0 (0)	6 (10)	0.0918^*^
Family planning services	19	129 (190)	161 (403)	0.6912	20	54 (72)	66 (76)	0.5195
HIV/AIDS/STI services								
STIs patients	20	43 (126)	94 (84)	0.1802	19	2.0 (6)	103 (107)	0.0005^**^
HIV tests for non-pregnant clients	24	55 (65)	104 (81)	0.0213^**^	21	97 (118)	101 (127)	0.8851
HIV tests for pregnant women	24	40 (43)	119 (128)	0.0067^**^	18	101 (117)	135 (143)	0.4551
HIV positive clients	19	3 (3)	2 (3)	0.0095^**^	19	2 (1.5)	2 (2)	0.6284
Patients on PMTCT	3	1 (1)	8 (13)	0.4240	4	26 (49)	1 (1)	0.3858
Patients on VCT	9	6 (6)	55 (81)	0.1164	8	1 (2)	62 (65)	0.0344^**^
Laboratory/radiological services								
Patients accessing X-ray	3	133 (231)	255 (241)	0.5696	3	0 (0)	120 (208)	0.4226
Ultrasounds	3	42 (18)	94 (114)	0.5219	8	21 (36)	22 (22)	0.9261
TB screenings	3	5 (4)	10 (16)	0.5596	8	26 (70)	5 (7)	0.3837
Malarial tests	5	43 (76)	380 (236)	0.0174^**^	7	0 (0)	294 (219)	0.0120^**^
General laboratory tests	5	81 (150)	576 (475)	0.0309^**^	7	0 (0)	611 (441)	0.0106^**^
General medical care services								
Circumcisions	15	3 (6)	8 (5)	0.0220^**^	14	4 (6)	11 (11)	0.0652^*^
Major surgical procedures	4	8 (4)	21 (19)	0.2153	5	1 (2)	6 (7)	0.1783
Minor surgical procedures	28	2 (4)	20 (21)	0.0000^***^	29	0 (1)	21 (18)	0.0000^***^

Source: Field Data Greater Accra and Western Regions (2014)

Legend: ^a^All means have been rounded up to the nearest decimal point; HIV (Human immune-deficiency virus); AIDS

(Acquired Immuno-deficiency Syndrome); STI (Sexually transmitted infection); PMTCT (Prevention of mother to child transmission); VCT (Voluntary

Counselling and testing); TB (Tuberculosis)

Paired t-test statistically significant at *p < 0.1; **p < 0.05; ***p < 0.0001

Records of five (5) intervention facilities that run tests for malaria showed that the average number of tests conducted per facility per month was 43 (SD = 76) at baseline but increased to 380 (SD = 236) at follow-up, *p* = 0.0174. Among the control facilities, the average number of malaria tests conducted per facility per month was 0 (SD = 0) at baseline but increased to 294 (SD = 219) at follow-up, *p* = 0.0120.

Furthermore, statistical test for predictive power of the intervention on the key outcome variables of interest showed that within the baseline and follow-up sub-samples, the community engagement intervention impacted positively on child immunizations (Coef. = 519.5, 95%CI = [− 599.5 to 1638.5]) and condom distribution (Coef. = 72.1, 95%CI = [− 143.6 to 287.1]). Other significant predictors of maternal and child health service utilization were facility ownership, region of clinic location, distance to nearest referral health facility and income level of clients. For instance, private health facilities were less likely to conduct spontaneous vaginal deliveries (SVDs) (Coef. = − 7.31, 95%CI = [− 15.70 to 1.08], *p* < 0.05), child immunizations (Coef = − 167.6, 95%CI = [− 313.8 to − 21.39], *p* < 0.01) and distribute condoms (Coef. = − 182.5, 95%CI = [− 279.2 to − 85.7], p < 0.05) (see Table 4).

**Table 4:** Multivariate multiple logistic regression analysis on the determinants of maternal, child and reproductive services

	Baseline data (2012)			Follow-up data (2014)		
	Dependent variables (Model 1)			Dependent variables (Model 2)		
	SVDs	Immunizations	Condom utilization	SVDs	Immunizations	Condom utilization
Independent variables	Coef.[(95% CI]	Coef.[(95% CI]	Coef.[(95% CI]	Coef.[(95% CI]	Coef.[(95% CI]	Coef.[(95% CI]
Intervention status						
CEI	−1.83[−9.40 5.74]	−44.87[− 176.8 87.07]	−2.75[−90.06 4.58]	−8.90[− 87.72 9.91]	519.5[−599.5 1638.5]	72.16[− 143.6 287.10]
No CEI	Ref	Ref	Ref	Ref	Ref	Ref
Ownership						
Private	−7.31[−15.70 1.08]^*^	−167.58[−313.8−21.39]^+^	− 182.5[− 279.2−85.7]*	15.61[− 120.6 51.8]	− 558.4[− 2492.4 375.5]	− 78.33[− 451.3 294.6]
Public	Ref	Ref	Ref	Ref	Ref	Ref
Location						
Rural	5.97[−1.95 13.89]	-75.89[-213.9 62.13]	−6.0[−97.4 85.3]	9.20[−83.81 102.2]	380.2[− 940.4 1700.8]	48.11[−206.6 302.8]
Urban	Ref	Ref	Ref	Ref	Ref	Ref
Region						
GAR	−10.36[−18.80−1.93]^+^	−231.18[−378.2−84.19]^+^	−66.4[− 163.7 30.9]	−40.17[− 240.0 159.7]	608.6[− 2228.9 3446.1]	101.8[− 445.4 648.9]
WR	Ref	Ref	Ref	Ref	Ref	Ref
Distance to nearest referral FH						
30–60 min	0.18[−0.00 0.37]^*^	1.01[−2.21 4.24]	0.76[−1.38 2.89]	.264 [1.88 2.41]	2.91[−27.58 33.41]	−0.21[−6.09 5.67]
> 60 min	Ref	Ref	Ref	Ref	Ref	Ref
Income group of clients						
Low income	13.55 [4.56 22.54]^+^	148.1[−8.56 304.8]^*^	45.76[−57.95 149.5]	40.8[− 155.4 237.0]	−692.9[− 3478.7 2092.8]	−71.78[− 608.10 465.4]
High income group	Ref	Ref	Ref	Ref	Ref	Ref
Staff capacity	−.007[−0.20 0.19]	− 0.50[−3.88 2.88]	−0.50[−2.74 1.73]	− 0.77[−4.99 3.45]	−4.82[−64.69 55.05]	− 0.86[−12.40 10.69]

Source: Field Data Greater Accra and Western Regions (2014); Legend: SVDs (Spontaneous vaginal deliveries); HF (Health Facility); ^*^p < 0.05; ^+^p < 0.01

Model 1 Equation: Outcome variable 1 (RMSE = 14.16; “R-sq” = 0.36; F = 4.35; *p* = 0.0007); Outcome variable 2 (RMSE = 246.70; “R-sq” = 0.28; F = 2.97; *p* = 0.01047); Outcome variable 3 (RMSE = 163.28; “R-sq” = 0.28; F = 2.93; *p* = 0.0114)

Model 2 Equation: Outcome variable 1 (RMSE = 40.30; “R-sq” = 0.32; F = 0.27; *p* = 0.9355); Outcome variable 2 (RMSE = 572.15; “R-sq” = 0.44; F = 0.45; *p* = 0.8327); Outcome variable 3 (RMSE = 110.34; “R-sq” = 0.31; F = .256; *p* = 0.9439)

Health facilities located in the Greater Accra region were less likely to conduct SVDs relative to health facilities located in the Western region (Coef. = − 10.36, 95%CI = [− 18.80 to − 1.93], p < 0.01), and likewise child immunizations (Coef. = − 231.18, 95%CI = [− 378.2 to − 84.19], p < 0.01) (see Table 4). Health facilities which were nearer to a referral hospital within ≤60 min travel time were more likely to record SVDs than facilities which were farther from a referral hospital of > 60 min of travel time (Coef. = 0.18, 95%CI = [− 0.00 to 0.37], p < 0.05) (see Table 4).

Apart from the core maternal and child health outcome indicators other general health service components that recorded significant improvement in intervention and control facilities over time were: number of general laboratory tests, male child circumcisions and minor surgical procedures per facility per month (see Table 4). Control facilities recorded more improvements than intervention facilities in terms of general laboratory tests, voluntary counselling and testing (VCT) for patients, number of patients treated with sexually transmitted infections (STIs), male child circumcisions, and other minor surgical procedures (see Table 4), suggesting the intervention perhaps had lesser impact on these service components. The researchers however, acknowledge extraneous factors other than the community engagement intervention which might have influenced the outcome of these indicators in the intervention and control facilities.

## Discussion

Engaging community members systematically to monitor the quality of healthcare services has the potential to enhance utilization of maternal and child healthcare services including child immunizations and condom distributions. It was observed that the community engagement intervention had a positive impact on HIV testing for pregnant women, thus corroborating similar previous studies in Ghana [11–16] and elsewhere [17] where evidence of bottom-up community-based intervention have influenced health outcomes including maternal and child health. Moreover, it was observed that intervention health facilities improved significantly in the number of clients tested for malaria before treatment from an average of 43 at baseline to 380 at follow-up and likewise general laboratory tests. These findings corroborate findings by Björkman & Svensson [18] in Uganda where community engagement positively influenced utilization of maternal and child health services in the study communities.

Similarly, Berlan and Shiffman [19] concluded that engaging communities and allowing them to participate in the service delivery process has the potential to promote health provider accountability to clients. As demonstrated in this study and similar studies by Alhassan et al. [5–8] on Ghana, a significant number of the maternal and child healthcare indicators improved in the intervention facilities as compared to the control facilities. It is important to however state that control facilities performed better than intervention facilities in terms of utilization of STIs services, VCT and minor surgical procedures, suggesting perhaps the intervention had no significant impact on these service components.

Findings of this study nonetheless, validate some literature on Ghana [20] and other African countries [21, 22] where client-centered healthcare system is advocated through bottom-up community engagement to promote acceptability and utilization of safer healthcare services. Previous studies have blamed the lack of clients’ confidence in formal healthcare systems on low level of engagement of communities in the entire value chain of healthcare delivery [18, 20].

Engagement of clients in health service delivery is more likely to enhance knowledge levels of mothers on importance of antenatal and postnatal care services as alluded by Asante-Sarpong et al. [20]. Similarly, a study by Agbozo et al. [16] underscored the positive association between a community-based health programme on child welfare, growth monitoring and mothers’ satisfaction level with service quality in a study on Ghana. Likewise, Afulani et al. [21] found a positive correlation between community-based approach to maternal healthcare and better service utilization among postnatal women in Western Kenya. Rurangirwa et al. [22] made similar conclusions in their study on antenatal service utilization in Rwanda.

Overall, findings of this study support some conclusions in a multi-country case study involving six countries including Ghana [23] which stated that multi-stakeholder engagement approaches including community engagement promotes interest and goodwill for nouvelle maternal and child health interventions in Africa. Generally, this cluster randomised trial demonstrates the critical role of community engagement in attainment of maternal and child health outcome indicators particularly at the primary healthcare level in Ghana.

## Limitations

First, it is imperative to acknowledge that there were factors outside the sphere of control of this study which were typically the human and material health resources of the study facilities. For instance, the study had no control of posting of new staff, transfer of old staff and general health infrastructure upgrade or deterioration throughout the baseline and follow-up periods. This limitation might have influenced utilization of maternal and child services independent of the community engagement intervention.

Also, this study focused mainly on primary health facilities in two (2) out of ten (10) administrative regions which might not reflect the situation in higher level health facilities and other regions. Future research endeavours should consider expanding the scope to include higher level facilities in more regions of Ghana to enhance national representativeness.

## Conclusion

This cluster randomised trial has demonstrated that bottom-up community engagement in health potentially improves utilization of key maternal and child health services such as normal deliveries, malarial tests and HIV tests for non-pregnant and pregnant mothers. However, the intervention did not significantly impact service components such as number of STIs and VCT services, male child circumcisions and other minor surgical procedures. The evidence suggests bottom-up community engagement in health promises to be a nouvelle strategy towards enhancing trust and confidence in low resource countries such as Ghana.

### Implications for public health policy and future research

1. The initial trial was implemented nearly seven (7) years ago, thus follow-up investigations on the continuity of the intervention by the Ghana Health Service (GHS) and National health Insurance Authority (NHIA) would inform strategies for reviving and sustaining this innovation.
2. There is need for policy dialogues on possibly replicating the bottom-up community engagement intervention in other regions and districts in Ghana as a basis for nationwide scale-up to complement existing efforts.
3. Following wider stakeholder consultations, the bottom-up community engagement innovation should be incorporated into existing peer reviews led by the GHS to enhance community participation in the service delivery value chain.

## Additional files

Additional file 1: Randomization and sampling procedure. Source: WOTRO-COHEiSION Ghana Project (2012–2014); Legend: GAR (Greater Accra); WR (Western Region); SCE (Systematic Community Engagement) (DOCX 33 kb)

Additional file 2: Systematic Community Engagement (SCE) implementation steps. Source: WOTRO-COHEiSION Ghana Project baseline and follow-up field data (2014), cited in Alhassan et al. (2015); Legend: C=Client; P=Provider; I=Insure; NHIS (National Health Insurance Scheme); NHIA (National Health Insurance Authority) (DOCX 30 kb)

Additional file 3: Overview of the Systematic Community Engagement (SCE) Interventions Implementation Steps. Source: WOTRO-COHEiSION Ghana Project, cited in Alhassan et al. (2015) (DOCX 13 kb)

## Data Availability

There are no restrictions to data sources per se but details of the full data may be accessed through the Principal Investigator (PI), Prof. Tobias F Rinke de Wit (Amsterdam Institute for Global Health and Development University of Amsterdam. Postal Address: Pietersberweg 17; 1105 BM Amsterdam. The Netherlands. E-mail: t.rindewit@aighd.org) and the Co-PI, Dr. Daniel Kojo Arhinful (Noguchi Memorial Institute for Medical Research, University of Ghana, Legon. PO Box LG 583, Legon. E-mail: DArhinful@noguchi.ug.edu.gh).

## Abbreviations

AIDS: Acquired-Immune Deficiency Syndrome
ANC: Antenatal Care
CHPS: Community-based Health Planning and Services
GAR: Greater Accra Region
GHS: Ghana Health Service
GMHS: Ghana Maternal Health Survey
HIV: Human Immunodeficiency Virus
LMICs: Lower Middle-Income Countries
MMR: Maternal Mortality Ratio
MoH: Ministry of Health
NHIA: National Health Insurance Authority
NHIS: National Health Insurance Scheme
OPD: Outpatient Department
PMTCT: Prevention of Mother to Child Transmission
SCE: Systematic Community Engagement
SDGs: Sustainable Development Goals
STI: Sexually Transmitted Infection
SVDs: Spontaneous Vaginal Deliveries
VCT: Voluntary Counselling and Testing
WHO: World Health Organization
WR: Western Region
