# Supplementary material for: Impact of a bottom-up community engagement intervention on maternal and child health services utilization in Ghana: a cluster randomised trial
Source: BMC Public Health. 2019 Jun 21;19:791. doi: 10.1186/s12889-019-7180-8 (PMC6588841; doi:10.1186/s12889-019-7180-8)
Supplement: Supplementary file 1 — Randomization and sampling procedure. Source: WOTRO-COHEiSION Ghana Project (2012–2014); Legend: GAR (Greater Accra); WR (Western Region); SCE (Systematic Community Engagement) (DOCX 33 kb) [file 12889_2019_7180_MOESM1_ESM.docx]

**Suppl. File 1:** Randomization and sampling procedure

**Source:** WOTRO-COHEiSION Ghana Project (2012-2014); **Legend:** GAR (Greater Accra); WR (Western Region); SCE (Systematic Community Engagement)
