# Supplementary material for: Impact of a bottom-up community engagement intervention on maternal and child health services utilization in Ghana: a cluster randomised trial
Source: BMC Public Health. 2019 Jun 21;19:791. doi: 10.1186/s12889-019-7180-8 (PMC6588841; doi:10.1186/s12889-019-7180-8)
Supplement: Supplementary file 2 — Systematic Community Engagement (SCE) implementation steps. Source: WOTRO-COHEiSION Ghana Project baseline and follow-up field data (2014), cited in Alhassan et al. (2015); Legend: C=Client; P=Provider; I=Insure; NHIS (National Health Insurance Scheme); NHIA (National Health Insurance Authority) (DOCX 30 kb) [file 12889_2019_7180_MOESM2_ESM.docx]

**Suppl. File 2:** Systematic Community Engagement (SCE) implementation steps

6 months

3 months

**Assessment areas**

=>Quality of services in NHIS-accredited clinics

**Source:** WOTRO-COHEiSION Ghana Project baseline and follow-up field data (2014), cited in Alhassan et al (2015); **Legend:** C=Client; P=Provider; I=Insure; NHIS (National Health Insurance Scheme); NHIA (National Health Insurance Authority)
