# Supplementary material for: Impact of a bottom-up community engagement intervention on maternal and child health services utilization in Ghana: a cluster randomised trial
Source: BMC Public Health. 2019 Jun 21;19:791. doi: 10.1186/s12889-019-7180-8 (PMC6588841; doi:10.1186/s12889-019-7180-8)
Supplement: Supplementary file 3 — Overview of the Systematic Community Engagement (SCE) Interventions Implementation Steps. Source: WOTRO-COHEiSION Ghana Project, cited in Alhassan et al. (2015) (DOCX 13 kb) [file 12889_2019_7180_MOESM3_ESM.docx]

**Suppl. File 3:** Overview of the Systematic Community Engagement (SCE) Interventions Implementation Steps

| The SCE intervention comprised of five steps that actively engaged clients in their communities to rate service quality in their nearest health facility using predefined quality service proxies. The five implementation steps are:  **Step 1:** Recruitment and training of 52 facilitators, and identification of existing community groups/associations. One facilitator was assigned to each of the of 52 community groups in the two study regions (26 in each region). Eligibility criteria for selection of community groups included: documented evidence of routine meetings (at least four times a year); regular meeting venue; clear leadership structure; non-partisan, and active membership not less than an intuitive number of ten (10). The community groups comprised of 22 religious/faith-based groups; 8 traders groups; 1 widows group; 3 community volunteers groups; 3 musician groups; 5 artisans groups and 11 youth groups. Average group size was 29 members (SD = 20).  **Step 2:** First round of assessment of service quality based on group members’ most recent (at most 6 months) experiences with the intervention service providers. Service quality indicators at healthcare provider level were: attitude of staff; punctuality of staff; availability of drugs; information provision; opportunity for feedback. Indicators for the health insurer are: information provision; (re)enrolment; delivering what is promised, and opportunity for feedback. A proxy indicator called Net Promotor Score (NPS) was used to measure clients’ trust for service providers. During the assessment, community members were asked to rank their experiences of service quality on a Five point Likert scale ranging from 1 “Very disappointing” to 5 “Very satisfactory”, using a community score card.  **Step 3:** Regional level validation and feedback sessions to disseminate the group assessment findings with facility heads, clients and NHIA representatives. This platform provided the service providers the opportunity to recognize and accept gaps in healthcare quality and agree on quality improvement plans with timelines and responsible persons.  **Step 4:** Follow-up on the service providers by facilitators (3 months after validation and feedback sessions) to ensure implementation of agreed action plans towards quality improvement.  **Step 5:** Rewarding best performing health facilities after a second round of community assessment (approximately six months after the first assessment). A citation plaque of honor and a token financial incentive of about US$ 280.00 was awarded best performing facilities to encourage competition among peers towards quality improvement. |
| --- |

**Source:** WOTRO-COHEiSION Ghana Project, cited in Alhassan et al (2015)
